# Consequences of age and education correction of cognitive screening tests – A simulation study of the MoCA test in Italy

**DOI:** 10.1007/s10072-024-07691-6

**Published:** 2024-07-16

**Authors:** Hans-Aloys Wischmann, Giancarlo Logroscino, Tobias Kurth, Marco Piccininni

**Affiliations:** 1 Institute of Public Health, Charité - Universitätsmedizin Berlin, Berlin, Germany; 2 Center for Neurodegenerative Diseases and the Aging Brain, Department of Clinical Research in Neurology, University of Bari “Aldo Moro”, “Pia Fondazione Cardinale G. Panico”, Tricase, Lecce Italy; 3 Department of Basic Medical Sciences, Neuroscience and Sense Organs, University of Bari “Aldo Moro”, Bari, Italy; 4 Digital Health - Machine Learning Research Group, Hasso Plattner Institute for Digital Engineering, Potsdam, Germany; 5 Digital Engineering Faculty, University of Potsdam, Potsdam, Germany

**Keywords:** Cognitive screening, Mild cognitive impairment, Dementia, Corrections, Age, Education

## Abstract

**Background:**

Cognitive screening tools are widely used in clinical practice to screen for age-related cognitive impairment and dementia. These tools' test scores are known to be influenced by age and education, leading to routine correction of raw scores for these factors. Despite these corrections being common practice, there is evidence suggesting that corrected scores may perform worse in terms of discrimination than raw scores.

**Objective:**

To address the ongoing debate in the field of dementia research, we assessed the impact of the corrections on discrimination, specificity, and sensitivity of the Montreal Cognitive Assessment test in Italy, both for the overall population and across age and education strata.

**Methodology:**

We created a realistic model of the resident population in Italy in terms of age, education, cognitive impairment and test scores, and performed a simulation study.

**Results:**

We confirmed that the discrimination performance was higher for raw scores than for corrected scores in discriminating patients with cognitive impairment from individuals without (areas under the curve of 0.947 and 0.923 respectively). With thresholds determined on the overall population, raw scores showed higher sensitivities for higher-risk age-education groups and higher specificities for lower-risk groups. Conversely, corrected scores showed uniform sensitivity and specificity across demographic strata, and thus better performance for certain age-education groups.

**Conclusion:**

Raw and corrected scores show different performances due to the underlying causal relationships between the variables. Each approach has advantages and disadvantages, the optimal choice between raw and corrected scores depends on the aims and preferences of practitioners and policymakers.

**Supplementary Information:**

The online version contains supplementary material available at 10.1007/s10072-024-07691-6.

## Introduction

Cognitive screening tests are brief psychometric instruments used in clinical practice to screen for cognitive impairment among previously undiagnosed persons or among individuals with subjective cognitive decline [1]. The aim of applying the test in this setting is to identify persons that have a high probability of suffering from cognitive impairment, for a timely referral to a neurologist and/or neuropsychologist for a comprehensive diagnostic workup and assessment. To discriminate individuals with cognitive impairment from those without, an individual’s test score is generally compared to an established cutoff that is predetermined to achieve a desired test performance in the target population [2].

Raw test scores from cognitive screening tests are routinely corrected for age and education [3, 4]. This demographic correction, sometimes referred to as “standardization” or “adjustment”, is applied so that a person’s test score is compared to the performance of “healthy” peers, i.e., individuals of same age and education, but without the condition of interest [5, 6]. However, the age-education correction of cognitive screening tests has been challenged in terms of statistical validity by both applied and methodological research [7–14]. Methodological criticisms were motivated by that fact that higher age and lower education are known to increase the probability of having cognitive impairment/dementia [7–9, 14]. More recently, findings have demonstrated that age-education correction diminishes the discrimination performance of the test, as measured by the area under the curve (AUC) of the receiver operating characteristic curve, when higher age and lower education both increase the probability of the condition and independently decrease the test performance [15]. However, discrimination performance is not the only relevant metric when evaluating a cognitive test in a real-world screening setting.

In this work, we therefore assessed the impact of the age-education correction on the discrimination performance (AUC), and on specificity and sensitivity for predetermined cutoffs, both overall and across age and education strata. We conducted a simulation study emulating a cognitive screening setting in Italy, using a realistic data generation model. Specifically, we focused on the consequences of age-education correction of the Montreal Cognitive Assessment (MoCA) test [16]. The choice of this particular case study was motivated by the clinical relevance, the availability of detailed information about the data generation process, and the fact that age-education correction has repeatedly been suggested for the MoCA test in Italy [17–20].

## Methods

### Model

To create a realistic data generation process, we used published information on the joint distribution of age and sex in the population, the conditional distribution of highest educational attainment by age and sex, the prevalence of mild cognitive impairment (MCI) and of dementia by age, sex, and education, and the distribution of MoCA test scores conditional on age, education, and cognitive status.

For the joint distribution of age and sex, we used data from the official reports on the Italian resident population as of January 1st, 2023 [21]. Our population of interest consisted of all residents aged between 55 and 89, as shown in Figure e1 in the Supplementary Information.

We extracted the proportion of residents that completed different levels of education by age group and sex from annual statistical reports (Annuario Statistico Italiano) published between 1998 and 2021 [22]. We converted the education levels into years of education by equating primary school (or less) with 5 years, middle school with 8, vocational qualification with 11, secondary education with 13, and university education with 17. This conversion is consistent with the number of school years in the education system in Italy and with mean values computed for the individuals tabulated in Aiello et al. (2022) [23]. As the proportion of individuals that completed a specific number of education years has changed over time due to socio-economic developments, policy changes, and modifications to the education system, we modeled the relationship between education and year of birth. For today’s elderly, relevant disparities in schooling access and education attainment existed between men and women when they were adolescents, so that we fitted local regression (loess) models by year of birth separately for men and women, as shown in Figure e2. We used all available data points across all publication years for the age groups from 30 to 64 years. The resulting models were used to approximate the distribution of years of education by year of birth and sex. Years of birth were then converted into age values, considering that our simulation was set into 2023. This procedure provided us with the conditional distribution of highest educational attainment by age and sex.

To model the prevalence of dementia and MCI in the population of interest by age, education, and sex, we used data from an older, population-based, cross-sectional study from Northern Italy [24], which evaluated the prevalence of dementia and of mild cognitive impairment in all persons above 60 years of age residing in two municipalities in Ravenna province. Here, we equated the category “6 or more years” of education with two completed levels of education (middle school), “4–5 years” with one completed level of education (primary school), and “1–3 years” as well as “no schooling” with zero levels. We fitted two separate Poisson regressions for the number of cases of dementia or MCI, with age (lower bound of the age interval plus 2), sex, and the number of completed levels of education as the independent variables, using the logarithm of the population size as the offset. The resulting prevalence ratios for dementia were 1.14 for each additional year of age (and thus 1.95 for each additional 5 years), 0.49 for each additional level of education, and 1.07 for female sex, while the ratios for MCI were 1.03 for each additional year of age (and thus 1.16 for each additional 5 years), 0.45 for each additional level of education, and 1.14 for female sex (see Table e1). These ratios are consistent with widely reported doubling of dementia prevalence for every five additional years of age, as also found in a systematic review and meta-analysis [25], and with previously reported protective effects of education. For MCI, the slower increase with age agrees with other previous findings [25]. The fitted regressions were then used to extrapolate the prevalences for all combinations of age, sex, and number of completed levels of education. However, the resulting marginal prevalences by age or by age and sex were much lower than recently reported, by approximately a factor of 2 for dementia [26], and by a factor of 6 for MCI [27]. We, therefore, increased the intercept to scale up the overall prevalence by a constant factor of 2 for dementia and 6 for MCI, leading to similar prevalences compared to the literature as shown in Tables e2 and e3. In doing so, we assumed that the number of diagnoses increased over the past 20 + years, without meaningfully altering the underlying prevalence ratios for age, sex, and education.

Finally, we combined the official distribution of the residents’ sex and age, the conditional distribution of education levels by sex and age, as well as the conditional distribution of cognitive status by sex, age, and education level to obtain the joint distribution of sex, age, completed education, and cognitive status. In evaluating the models for the prevalence of MCI and dementia, the categories vocational training (11 years) and secondary education (13 years) from the annual reports were both mapped to three completed levels of education, and university education (17 years) to four completed levels of education. The resulting joint distribution represented our population of interest and it is reported in Table 1.

**Table 1:** Joint distribution of sex, age, completed education, and cognitive status (healthy, mild cognitive impairment (MCI), and dementia (Dem)) for the simulated population of Italian residents aged 55 to 89 years, as of January 1st, 2023. Each cell represents the probability (in %) of sampling an individual with a specific value of sex, age, education, and cognitive status

Sex	Age	Completed Education														
		5 years / 1 level			8 years / 2 levels			11 years / 3 levels			13 years / 3 levels			17 years / 4 levels		
		Healthy	MCI	Dem	Healthy	MCI	Dem	Healthy	MCI	Dem	Healthy	MCI	Dem	Healthy	MCI	Dem
f	55–59	0.660	0.100	0.004	3.955	0.244	0.012	0.929	0.025	0.001	3.491	0.093	0.005	1.412	0.017	0.001
f	60–64	0.972	0.175	0.013	3.329	0.240	0.020	0.822	0.025	0.002	2.790	0.086	0.008	1.105	0.015	0.001
f	65–69	1.537	0.334	0.043	2.581	0.219	0.030	0.620	0.022	0.003	1.993	0.072	0.011	0.937	0.015	0.002
f	70–74	2.266	0.608	0.131	1.995	0.202	0.047	0.446	0.019	0.005	1.347	0.057	0.014	0.734	0.014	0.004
f	75–79	2.407	0.817	0.289	1.386	0.167	0.064	0.278	0.014	0.006	0.834	0.041	0.017	0.447	0.010	0.004
f	80–84	2.133	1.015	0.609	0.916	0.138	0.090	0.153	0.009	0.006	0.508	0.030	0.022	0.229	0.006	0.004
f	85–89	1.115	0.911	0.903	0.450	0.088	0.094	0.055	0.004	0.005	0.259	0.019	0.022	0.066	0.002	0.002
m	55–59	0.567	0.073	0.003	4.396	0.236	0.012	0.755	0.018	0.001	3.197	0.075	0.004	1.224	0.013	0.001
m	60–64	0.654	0.101	0.008	3.557	0.223	0.019	0.609	0.016	0.002	2.695	0.073	0.007	1.040	0.012	0.001
m	65–69	0.940	0.175	0.024	2.682	0.198	0.029	0.474	0.015	0.002	2.135	0.067	0.011	0.922	0.013	0.002
m	70–74	1.392	0.317	0.073	2.085	0.183	0.045	0.371	0.014	0.004	1.594	0.059	0.016	0.773	0.012	0.004
m	75–79	1.500	0.426	0.160	1.420	0.148	0.060	0.235	0.010	0.005	1.019	0.044	0.020	0.523	0.010	0.005
m	80–84	1.311	0.509	0.325	0.876	0.113	0.078	0.122	0.006	0.005	0.563	0.029	0.022	0.304	0.007	0.006
m	85–89	0.665	0.411	0.431	0.380	0.062	0.071	0.036	0.002	0.003	0.205	0.013	0.016	0.114	0.003	0.004

f = female; m = male

From normative studies, we identified four published models for predicting the average raw MoCA test scores for Italians without cognitive impairment [17–20]:

1 $${\widehat{MoCA}}_{Aiello}=24.17-0.000008*\left({age}^{3}-297697.18\right)+3.331407*\left(\text{ln}\left(edu\right)-2.325648\right)$$

2 $${\widehat{MoCA}}_{Conti}=23.28-0.175*\left(age-70.08\right)-24.3*\left(1/edu-0.126\right)$$

3 $${\widehat{MoCA}}_{Santangelo}=21.98+4.228*\left({\text{log}}_{10}\left(100-age\right)-1.58\right)+3.201*\left(\sqrt{edu}-3.25\right)$$

4 $${\widehat{MoCA}}_{Montemurro}=25.468-0.089*\left(age-67.086\right)+0.187* \left(edu-11.245\right)$$

Since Montemurro et al. provided several models for raw scores as a function of different combinations of sex, age, and education, the parameters in Eq. (4) were computed from fitting a linear regression on the publicly available data from their study [20]. For patients with MCI and with dementia, the average raw MoCA scores were calculated by subtracting 5.1 and 10.7 points, respectively. These values were obtained as the rounded averages of a) differences of -5.333 ± 0.531 and -12.278 ± 0.592 between mean MoCA scores in a study in Portugal where MCI, dementia, and control subgroups were matched on age and education [28], b) coefficients of -4.07 ± 0.63 and -9.66 ± 0.84 from the combined regression model including age and education (in addition to sex, years in the US, and primary language) in a study of monolingual Chinese Americans [29], and c) coefficients of -5.769 ± 0.696 and -10.147 ± 0.688 from the combined regression model including only age, years of education, and clinical diagnosis in a study in Hong Kong [30]. These effects were comparable to: mean differences of 5.44 and 8.77 found in a study in Italy, where groups were not matched by age nor education [31] as well as mean differences of 5.20 for probable MCI patients with MMSE ≤ 23.8 compared to matched healthy controls with MMSE > 23.8 and mean differences of 9.45 or 10.55 for Dementia patients compared to two different groups of matched healthy controls, in a small study in Italy [32].

Raw MoCA scores were assigned to each person by using the mean MoCA test score given their age, education, and cognitive status, as obtained from Eq. (1) for the main analysis, plus a normally distributed error representing the influence of unobserved variables. We assumed errors to be independent and identically distributed with a mean of 0 and a standard deviation of 2.9 (standard deviation of the residuals obtained after fitting the regression model to the original data in Aiello et al. [23]).

### Simulation and data analysis

Using the data generation mechanism described above, we simulated a development sample of 5,000 persons and a separate validation sample of 50,000 persons. All individuals were independently drawn from a near-infinite super-population with the joint distribution of sex, age-group, education, and cognitive status shown in Table 1. Age was then assigned as a continuous value uniformly randomly drawn within the respective age-group limits, and raw MoCA scores were generated according to Eq. (1).

We then fitted a regression with the raw MoCA test scores as the dependent variable and age and education as the independent variables only among the “healthy” individuals (without cognitive impairment) from the development sample, using the same terms for age and education as in Eq. (1). Using the resulting predictions (${\widehat{MoCA}}_{prediction}$) and the intercept of the model (${\beta }_{0}$), we computed corrected scores for all individuals according to Eq. (5), without rounding or clipping. This approach is traditionally used to correct Italian neuropsychological tests [33, 34], and commonly employed to correct MoCA scores for age and education [17–19]. The corrected score for an individual *A* is the difference between (i) the observed raw score for individual *A* and (ii) the expected raw score for a healthy individual of same age and education as individual *A*, plus (iii) a constant to ensure that the mean score for the population of healthy individuals remains unchanged.

5 $${Corrected}_{A}={Raw}_{A}- {\widehat{MoCA}}_{prediction}\left(age={age}_{A}, education={education}_{A}\right)+{\beta }_{0}$$

This approach results in the same AUC, sensitivity, and specificity that would be obtained using the common Z-score correction [5], as these metrics are invariant to additive shifts and the standard deviation of the residuals is constant (so that dividing by it would not alter ranks) [15].

We then evaluated the overall discrimination performance in the validation sample, estimating the AUC, for both raw scores and for corrected scores, and the AUC difference. AUC values were computed to measure the discrimination performance for distinguishing individuals with cognitive impairment (MCI or dementia) from those without, and separately for distinguishing individuals with MCI from those without cognitive impairment (excluding patients with dementia). As the latter task reflects most closely the scenario of screening previously undiagnosed persons for cognitive impairment, sensitivity and specificity were estimated for this contrast only.

To compute sensitivity and specificity, it is necessary to determine cutoffs a priori for corrected and for raw scores. We determined these cutoffs marginally, i.e., based on their performance on the overall population, in the development sample. This choice was motivated by the fact that raw scores and corrected scores are expected to lead to identical sensitivities and specificities if individual cutoffs were instead determined for each age-education group (which would implicitly correct for age and education).

Many options exist to select cutoffs for cognitive screening tests. It is common practice to choose the cutoff for corrected scores by considering a sample of “healthy” individuals with the same demographic characteristics and selecting the value corresponding to the mean score minus 1 or 2 standard deviations. Under the assumption of normality and constant variance of the residuals, this technique is equivalent to choosing the cutoffs corresponding to a specificity of 84.1% and 97.7%. Coherently, we determined the cutoffs in the development sample as the values that ensured a marginal preselected specificity of either 97.7% or 84.1%. In view of the importance of sensitivity in a screening setting as also highlighted in [35], a third cutoff was determined from the scores of the MCI patients in the development sample, preselecting a sensitivity of 84.1%. Sensitivity and specificity were calculated in the validation sample, both marginally and by age-education group, separately for raw scores and for corrected scores.

The above simulation procedure was repeated 10,000 times, and means were computed together with 2.5th and 97.5th percentiles for all metrics. In the main analysis, MoCA scores were generated and corrected according to Eq. (1) and neither rounded nor clipped. To assess the robustness of the results, we conducted sensitivity analyses, in which MoCA scores were computed using each of the Eqs. (1) to (4), rounded to the nearest integer, and truncated to the valid range of test scores, i.e., [0,30] points. Furthermore, we repeated the main analysis using a larger standard deviation of 3.4 for the residuals.

All simulations and evaluations were performed using RStudio version 2024.04.1 Build 748 [36], R version 4.4.0 [37], and R package pROC version 1.18.4 [38].

## Results

Table 2 presents the average AUC values for corrected and raw MoCA scores and their differences. The AUC values for raw scores were higher than for corrected scores, both in discriminating patients with cognitive impairment (MCI or dementia) from “healthy” individuals (mean AUC of 0.9465 [0.944,0.949] for raw scores, mean difference of 0.0233 [0.020,0.027]), and in discriminating MCI patients from individuals without cognitive impairment (mean AUC of 0.9251 [0.921,0.929] for raw scores, mean difference of 0.0320 [0.027,0.037]).

**Table 2:** Average Area Under the Curve (AUC) in the validation sample across 10,000 simulations from the main analysis. The AUC is presented for raw and corrected scores, along with their difference, for discriminating patients with cognitive impairment (Mild Cognitive Impairment or Dementia) from Healthy individuals, and for discriminating patients with Mild Cognitive Impairment from Healthy individuals (excluding Dementia cases). The 2.5th and 97.5th percentiles are also reported

Scores	(MCI or Dem.) vs. Healthy	MCI vs. Healthy
Raw	0.9465 [0.944,0.949]	0.9251 [0.921,0.929]
Corrected	0.9232 [0.918,0.928]	0.8931 [0.887,0.899]
Difference	0.0233 [0.020,0.027]	0.0320 [0.027,0.037]

When raw MoCA scores were rounded and clipped in the sensitivity analysis using Eq. (1), the corresponding AUC mean differences for these two contrasts were 0.0218 [0.018, 0.026] and 0.0300 [0.025, 0.035], respectively, again in favor of the raw scores, as shown in Table e4. Similar results, with mean AUC differences ranging from 0.0171 to 0.0225 and from 0.0234 to 0.0313 were also observed in the other sensitivity analyses using Eqs. (2) to (4), as reported in Tables e5 to e7. When the main simulation was repeated with a standard deviation of residuals of 3.4, the AUC difference for both discrimination tasks was even more pronounced, as shown in Table e8.

Table 3 shows the sensitivity and specificity for discriminating MCI patients from persons without cognitive impairment for the main analysis, while the corresponding results for the sensitivity analyses are provided in Tables e9-e12 in the Supplementary Information.

**Table 3:** Average sensitivity and specificity in the validation sample across 10,000 simulations from the main analysis. Values are presented for raw and for corrected scores, for discriminating Mild Cognitive Impairment patients from healthy individuals. The 2.5th and 97.5th percentiles are also reported. The upper part shows the average sensitivity and specificity for the total validation sample, using three different cutoffs derived marginally from the development samples. The lower parts report the respective values of sensitivity and specificity for the two extreme age-education strata, i.e., the youngest with the highest education and the oldest with the lowest education

		Raw		Corrected		
Age	Edu	Sensitivity [%]	Specificity [%]	Sensitivity [%]	Specificity [%]	Cutoff
*	*	51.6 [48.0,55.1]	97.7 [97.2,98.1]	40.6 [36.9,44.3]	97.7 [97.2,98.1]	Specificity = 97.7%
		85.1 [83.6,86.5]	84.1 [83.0,85.2]	77.6 [75.5,79.6]	84.1 [82.9,85.2]	Specificity = 84.1%
		84.0 [80.4,87.4]	85.1 [81.6,88.1]	84.0 [80.4,87.3]	77.6 [73.2,81.6]	Sensitivity = 84.1%
(54,59]	17	4.1 [0.0,16.7]	100.0 [99.9,100.0]	40.6 [15.4,66.7]	97.7 [96.7,98.6]	Specificity = 97.7%
(84,89]	5	81.7 [77.6,85.6]	80.3 [76.4,84.1]	40.6 [34.9,46.4]	97.7 [96.4,98.8]	
(54,59]	17	30.2 [7.7,55.6]	98.9 [98.2,99.4]	77.5 [53.8,100.0]	84.1 [81.5,86.6]	Specificity = 84.1%
(84,89]	5	98.4 [97.3,99.3]	35.2 [31.5,39.0]	77.6 [73.3,81.6]	84.1 [80.7,87.2]	
(54,59]	17	28.5 [6.7,53.8]	99.0 [98.2,99.6]	83.9 [62.5,100.0]	77.6 [72.1,82.6]	Sensitivity = 84.1%
(84,89]	5	98.1 [96.7,99.2]	37.3 [30.3,44.5]	84.0 [79.4,88.4]	77.6 [72.2,82.6]	

The average sensitivity in the whole validation sample was higher for raw scores (51.6% and 85.1%) than for corrected scores (40.6% and 77.6%), at identical specificity levels of 97.7% and 84.1%, respectively. Similarly, the average specificity was higher for raw scores (85.1%) than for corrected scores (77.6%), at an identical sensitivity level of 84.1%.

When using raw scores for the cutoff that ensures a marginal specificity of 84.1% across the whole sample, the average sensitivity varied substantially across the age-education strata (from 30.2% in the youngest with the highest education to 98.4% in the oldest, with the least education). For the same cutoff, the specificity also varied substantially (from 35.2% in the oldest with the least education to 98.9% in the youngest with the highest education). This strong heterogeneity in the age-education-specific performances was also found for the other cutoffs that ensure a marginal sensitivity of 84.1% or a marginal specificity of 97.7%. For the latter, the average sensitivity for the youngest with the highest education was only 4.1%, while the average specificity reached 100%. By contrast, for each cutoff, when using corrected scores, sensitivity and specificity remained virtually identical across the age-education groups. Similar patterns were observed in the sensitivity analyses.

**Fig. 1:**
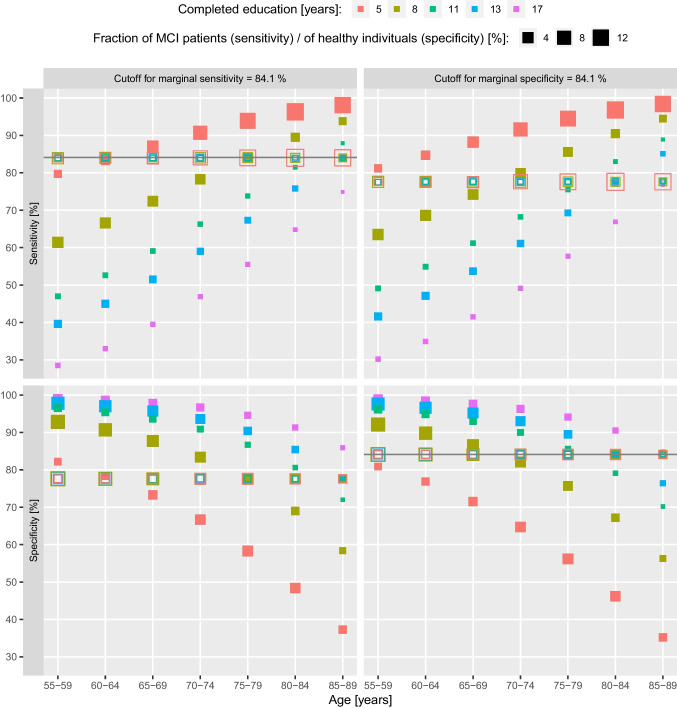
Average sensitivity (top) and specificity (bottom) for discriminating MCI patients from healthy individuals in the validation sample across 10,000 simulation runs in the main analysis, showing results for all age-education strata. Values for raw MoCA scores are depicted as filled symbols, while corrected MoCA scores are depicted as hollow symbols. The size of each square corresponds to the fraction of MCI patients (top) or of healthy individuals (bottom) in the respective age-education group. The cutoff for a marginal sensitivity of 84.1% was used for the left panels, while the cutoff for a marginal specificity of 84.1% was used for the right panels, as denoted by the horizontal lines

The different behaviors of corrected and raw scores can easily be understood from Fig. 1, where average sensitivity and specificity are shown for all age-education groups. For a fixed marginal specificity, the raw scores showed higher sensitivity in the higher-risk groups (older / lower education) and lower sensitivity in the lower-risk groups (younger / higher education) compared to the corrected scores. At the same time, for a fixed marginal sensitivity, the raw scores showed higher specificity in the lower-risk groups, and lower specificity in the higher-risk groups, compared to the corrected scores.

The marginal sensitivity (or marginal specificity) is simply the weighted average of the age-education-specific sensitivity (or specificity), with weights equal to the proportion of individuals in the age-education group among MCI patients (or healthy individuals). Therefore, higher-risk groups contribute more to the marginal sensitivity and less to the marginal specificity, compared to lower-risk groups. When choosing the cutoff that ensures a marginal specificity of 84.1%, the marginal sensitivity for the corrected scores was 77.6%, equal to the sensitivity for each age-education group (Fig. 1), but the marginal sensitivity for the raw scores was higher (85.1%). This is because the majority of MCI patients belonged to age-education groups in which the raw score sensitivity was higher (see top right of Fig. 1). A similar explanation can be given for the specificity metric when using a marginal cutoff for sensitivity.

## Discussion

In a simulation that closely reflects a real-world setting of age-related cognitive impairment and dementia screening in Italian residents aged 55 to 89, our study confirmed that the discrimination performance of the MoCA test is higher for raw scores than for scores that are corrected for age and education, both in distinguishing patients with MCI or dementia from individuals without cognitive impairment and in distinguishing MCI patients from individuals without cognitive impairment (in the absence of patients with dementia), consistent with analytical results [15]. In a recent Italian study, Ilardi et al. compared the discrimination performances of the raw MoCA score (with the traditional + 1 correction for education) with three different regression-based age-education corrections [35]. The authors did not find a statistically significant difference between the four AUCs. However, their study included only 45 patients (21 early stage dementia and 24 MCI) and 25 healthy controls [35].

We also investigated sensitivity and specificity for raw and corrected scores, in distinguishing MCI patients from individuals without cognitive impairment for three different cutoffs. Our comparison revealed that raw scores demonstrated superior sensitivity in the overall population, when specificity was held constant, and superior specificity in the overall population when sensitivity was fixed. However, we observed substantial variability in both sensitivity and specificity among different age-education groups for raw scores, with very low sensitivities and specificities observed for some groups. In contrast, corrected scores demonstrated the same performances across all demographic groups.

While perhaps counterintuitive, these results are easily explained by the causal effects at play in our assumed data generating process. The disparate sensitivities and specificities of the raw test scores across the different age-education groups are a consequence of the direct influence of age and education on test performance: higher age and lower education negatively impact the raw test scores (both in patients with and without cognitive impairment). For a fixed cutoff at the overall population level, the raw scores, therefore, have a higher sensitivity among older and less educated individuals, and a higher specificity among younger and more educated individuals.

On the other hand, the prevalence of cognitive impairment increases with higher age and lower education, so that the age-education-specific sensitivities (or specificities) contribute with different weights to the sensitivity (or specificity) in the overall population. Younger, highly educated individuals contribute more to the specificity of the overall population, while older, less educated individuals contribute more to the sensitivity of the overall population. Consequently, raw test scores showed better overall performances compared to corrected scores at the chosen cutoffs: raw scores have better sensitivity and specificity in the age-education groups that contribute more to the respective overall performances. The age-education correction removes the impact of age and education on the test score. Therefore, the sensitivities (and specificities) become constant across all age-education groups after the correction.

Both approaches have advantages and disadvantages, so that the choice between raw or corrected scores depends on the aims and preferences of practitioners and policymakers. Based on these aims and preferences, costs and utility values are assigned to (i) correctly identified MCI cases (true positives), (ii) missed MCI cases (false negatives), (iii) unnecessary referrals (false positives), and (iv) correctly identified healthy individuals (true negatives), overall and for each demographic group. These costs and utility values weigh the advantages and disadvantages of the two approaches, and should drive the choice for one or the other. For instance, when we assign uniform utilities or costs to the detection of MCI cases, irrespective of the individual's age or education (meaning the value of diagnosing a 55-year-old with MCI is considered equivalent to diagnosing an 85-year-old), raw scores emerge as the preferable option. This is because they enhance the detection rate of MCI cases across the entire population at a specified level of specificity. Conversely, if the goal is to guarantee that individuals of any age or education level receive equal treatment in terms of sensitivity and specificity, corrected scores become the preferred choice. In the context of “algorithmic fairness” literature, applying age-education correction fulfills the fairness criterion of “equalized odds” across different age and education groups [39].

We emphasize that this decision has direct impact on patient care. While the issue of age-education correction might appear intuitive and simple, it can be difficult to grasp the impact of age-education correction (or lack thereof) purely relying on intuition, due to the danger of conflating causal and prediction tasks. We, therefore, caution against implementing changes in clinical care without careful consideration of the relevant ethical, clinical, and statistical aspects.

### Limitations and future work

The development sample in each simulation run was chosen to mimic a very large study with approximately 5,000 individuals that are representative of the target population in terms of sex, age, education, and cognitive status. We realize that individual studies with these characteristics are rare for the purpose of establishing norms or cutoffs for cognitive screening tests. Moreover, we chose to reproduce the scenario of cognitive screening in a population setting. Our results may thus not be transportable to other settings, where the associations and the distribution of demographic/clinical characteristics may differ.

We built a data generation process for the chosen case study using available, published data. As the evidence was limited for some distributions, we needed to make simplifying approximations. For example, we assumed that the difference in test scores between dementia patients, MCI patients, and individuals without cognitive impairment were similar across study geographies and language versions of the MoCA test, after adjusting for age and education. Moreover, no recent data for the prevalence of dementia and MCI by age and education was available for the population of interest, so that we built our model using data from De Ronchi et al. [24]. We reported all our choices in the methods section and, where possible, ran sensitivity analyses to evaluate the robustness of the findings under different assumptions and for different regression models.

In our analyses, we considered three possible ways to obtain cutoffs: two based on specificity in a normative sample, as it is common clinical practice; and one based on sensitivity, using information on the test score distribution among individuals with the medical condition. We recognize that other strategies are possible, and that this is the at the center of methodological debates [35]. We emphasize, however, that age-education correction is a separate issue as indicated by the difference in the AUCs, a metric that summarizes sensitivity and specificity across all possible cutoffs.

We have quantified and described the impact of using raw vs. corrected scores in terms of well-known metrics, including the AUC as well as sensitivity and specificity. We did not explicitly consider in our simulation other important metrics such as the False Omission Rate (as the complement of the Negative Predictive Value) or the Positive Predictive Value, which may be also highly informative.

Over time, changes in the age distribution (aging population) and in the shares of individuals that completed different education levels can shift the distribution of raw test scores, and thereby shift the cutoffs that ensure a preselected specificity or sensitivity. On the other hand, if the conditional distribution of the test score given age, education, and cognitive status does not change over time, the distribution of the corrected scores will not change, and the performance of established cutoffs will remain stable. Future work could compare the temporal validity of raw and corrected scores in real-world data.

## Conclusion

In our simulation of the MoCA test in a screening setting in Italy, the discrimination performance of the test was confirmed to be slightly higher using raw scores compared to using scores that are corrected for age and education. For fixed cutoffs, raw scores exhibited higher sensitivity in higher-risk groups and higher specificity in lower-risk groups, explaining the overall superiority. Raw scores showed very heterogenous performances, with poor sensitivities and specificities in certain demographic groups. On the contrary, corrected scores showed homogeneous sensitivities and specificities across all age-education strata. The different behaviors are explained by the causal relationships between the test score, cognitive status, and demographic variables. The choice between raw and corrected scores should be motivated by explicit consideration of the physician’s aims and preferences for the cognitive screening tasks at hand.

## Supplementary Information

Below is the link to the electronic supplementary material.

Supplementary file1 (DOCX 1.96 MB)

## Data Availability

All the data analyzed in this article were simulated, and the code to reproduce the simulation can be found on github at https://github.com/wischmha/Cognitive-Screening-Simulation
