# Supplementary material for: Consequences of age and education correction of cognitive screening tests – A simulation study of the MoCA test in Italy
Source: Neurol Sci. 2024 Jul 16;45(12):5697–706. doi: 10.1007/s10072-024-07691-6 (PMC11554764; doi:10.1007/s10072-024-07691-6)
Supplement: Supplementary file 1 — Supplementary file1 (DOCX 1.96 MB) [file 10072_2024_7691_MOESM1_ESM.docx]

**Supplementary Information**

**Journal**

Neurological Sciences

**Title**

Consequences of Age and Education Correction of Cognitive Screening Tests – A Simulation Study of the MoCA Test in Italy

**Authors**

Hans-Aloys Wischmann^1^, Giancarlo Logroscino^2,3^, Tobias Kurth^1^, Marco Piccininni^1,4,5^

^1^ Institute of Public Health, Charité - Universitätsmedizin Berlin, Germany,

^2^ Center for Neurodegenerative Diseases and the Aging Brain, Department of Clinical Research in Neurology, University of Bari "Aldo Moro", "Pia Fondazione Cardinale G. Panico", Tricase, Lecce, Italy

^3^ Department of Basic Medical Sciences, Neuroscience and Sense Organs, University of Bari "Aldo Moro", Bari, Italy

^4^ Digital Health - Machine Learning Research Group, Hasso Plattner Institute for Digital Engineering, Potsdam, Germany

^5^ Digital Engineering Faculty, University of Potsdam, Potsdam, Germany

**Current address of corresponding author:**

Marco Piccininni, PhD

Hasso Plattner Institute for Digital Engineering

Campus Griebnitzsee, Universität Potsdam

Prof.-Dr.-Helmert-Straße 2 -3

14482 Potsdam

marco.piccininni@hpi.uni-potsdam.de


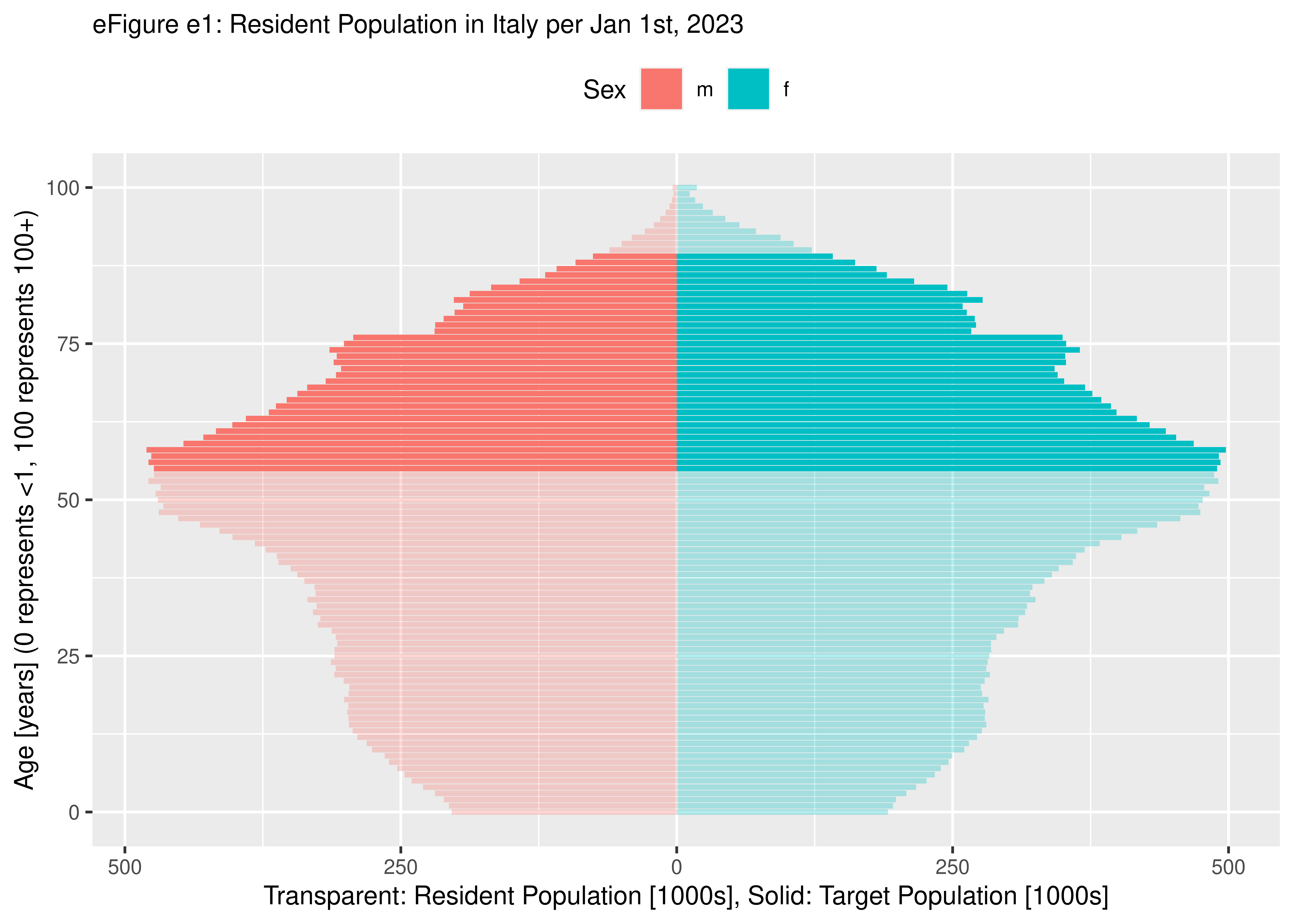


*Figure e1: Resident population in Italy as of January 1st, 2023. The plot shows the joint distribution of sex and age from the official ISTAT report* [1]*. The solid bars highlight the population of interest for this study, consisting of all residents between 55 and 89 years of age.*


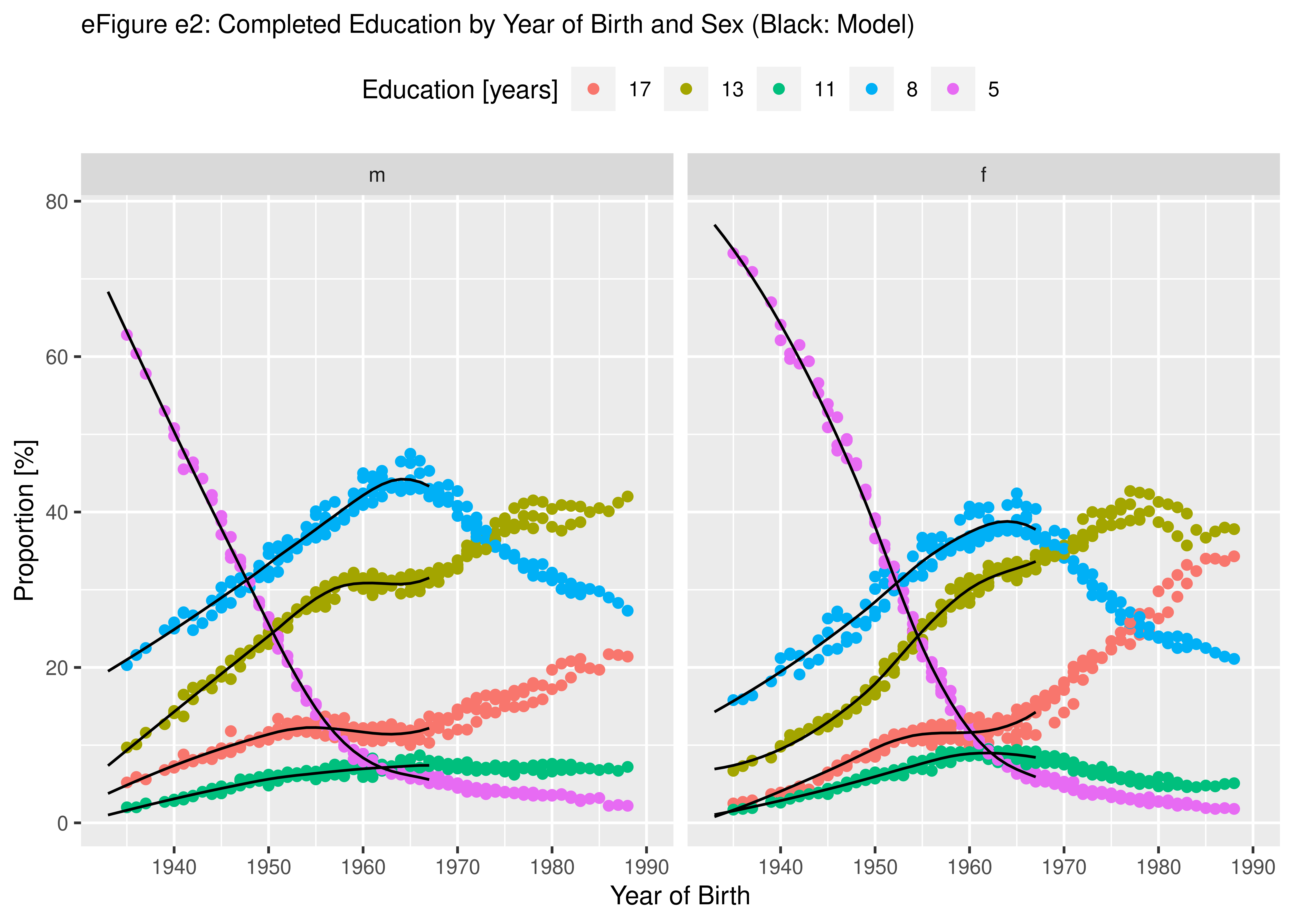


*Figure e2: Completed education for the resident population of Italy from 55 to 89 years of age in 2023, by sex and year of birth, showing data from the annual statistical reports (Annuario Statistico Italiano) published between 1998 and 2021* [2]*. The black lines show the model for the proportion of each education level, aggregated across the available data points using local regression (loess).*

*Table e1: Prevalence ratios (PR) for one additional year of age, one additional level of education, and female sex, from separate Poisson regression models for the prevalence of dementia and of MCI. Regressions were fit to the data by age and education groups from De Ronchi et al. (2005)* [3]*.*

| Coefficient | PR_Dementia | PR_MCI |
| --- | --- | --- |
| Age | 1.143 [1.129,1.157] | 1.030 [1.017,1.042] |
| Education level | 0.493 [0.416,0.580] | 0.447 [0.373,0.532] |
| Female sex | 1.068 [0.884,1.295] | 1.139 [0.923,1.412] |

MCI = mild cognitive impairment

*Table e2: Prevalence of MCI by age estimated from the model compared to published data from the US* [4]*.*

|  | Age | Prevalence [%] | | |
| --- | --- | --- | --- | --- |
|  |  | Model | US | Difference |
|  | 55-59 | 4.1 |  |  |
|  | 60-64 | 5.2 | 6.7 | -1.5 |
|  | 65-69 | 7.0 | 8.4 | -1.4 |
|  | 70-74 | 10.0 | 10.1 | -0.1 |
|  | 75-79 | 13.6 | 14.8 | -1.2 |
|  | 80-84 | 18.3 | 25.2 | -6.9 |
|  | 85-89 | 23.6 |  |  |

MCI = mild cognitive impairment

*Table e3: Prevalence of dementia by sex and age estimated from the model compared to published data from Europe* [5]*.*

| Sex | Age | Prevalence [%] | | |
| --- | --- | --- | --- | --- |
|  |  | Model | Europe | Difference |
| f | 55-59 | 0.2 |  |  |
| f | 60-64 | 0.5 | 0.9 | -0.4 |
| f | 65-69 | 1.1 | 1.5 | -0.4 |
| f | 70-74 | 2.5 | 3.4 | -0.9 |
| f | 75-79 | 5.6 | 8.9 | -3.3 |
| f | 80-84 | 12.5 | 13.1 | -0.6 |
| f | 85-89 | 25.7 | 24.9 | 0.8 |
| m | 55-59 | 0.2 |  |  |
| m | 60-64 | 0.4 | 0.2 | 0.2 |
| m | 65-69 | 0.9 | 1.1 | -0.2 |
| m | 70-74 | 2.0 | 3.1 | -1.1 |
| m | 75-79 | 4.5 | 7.0 | -2.5 |
| m | 80-84 | 10.2 | 10.7 | -0.5 |
| m | 85-89 | 21.7 | 16.3 | 5.4 |

f= female, m = male

*Table e4: Average Area Under the Curve (AUC) in the validation sample across 10,000 simulations from the sensitivity analysis using the model from Equation 1 with rounding and clipping. The AUC is presented for raw and corrected scores, for discriminating patients with cognitive impairment (Mild Cognitive Impairment or Dementia) from Healthy individuals, and for discriminating patients with Mild Cognitive Impairment from Healthy individuals (excluding Dementia cases). The 2.5^th^ and 97.5^th^ percentiles are also reported.*

| Scores | (MCI or Dem.) vs. Healthy | MCI vs. Healthy |
| --- | --- | --- |
| Raw | 0.9455 [0.943,0.948] | 0.9238 [0.920,0.928] |
| Corrected | 0.9237 [0.919,0.928] | 0.8937 [0.887,0.900] |
| Difference | 0.0218 [0.018,0.026] | 0.0300 [0.025,0.035] |

*Table e5: Average Area Under the Curve (AUC) in the validation sample across 10,000 simulations from the sensitivity analysis using the model from Equation 2 with rounding and clipping. The AUC is presented for raw and for corrected scores, for discriminating patients with cognitive impairment (Mild Cognitive Impairment or Dementia) from Healthy individuals, and for discriminating patients with Mild Cognitive Impairment from Healthy individuals (excluding Dementia cases). The 2.5^th^ and 97.5^th^ percentiles are also reported.*

| Scores | (MCI or Dem.) vs. Healthy | MCI vs. Healthy |
| --- | --- | --- |
| Raw | 0.9414 [0.938,0.944] | 0.9181 [0.914,0.922] |
| Corrected | 0.9243 [0.920,0.929] | 0.8946 [0.889,0.901] |
| Difference | 0.0171 [0.013,0.021] | 0.0234 [0.018,0.028] |

*Table e6: Average Area Under the Curve (AUC) in the validation sample across 10,000 simulations from the sensitivity analysis using the model from Equation 3 with rounding and clipping. The AUC is presented for raw and for corrected scores, for discriminating patients with cognitive impairment (Mild Cognitive Impairment or Dementia) from Healthy individuals, and for discriminating patients with Mild Cognitive Impairment from Healthy individuals (excluding Dementia cases). The 2.5^th^ and 97.5^th^ percentiles are also reported.*

| Scores | (MCI or Dem.) vs. Healthy | MCI vs. Healthy |
| --- | --- | --- |
| Raw | 0.9452 [0.942,0.948] | 0.9237 [0.920,0.928] |
| Corrected | 0.9227 [0.918,0.927] | 0.8925 [0.886,0.898] |
| Difference | 0.0225 [0.019,0.027] | 0.0313 [0.026,0.037] |

*Table e7: Average Area Under the Curve (AUC) in the validation sample across 10,000 simulations from the sensitivity analysis using the model from Equation 4 with rounding and clipping. The AUC is presented for raw and for corrected scores, for discriminating patients with cognitive impairment (Mild Cognitive Impairment or Dementia) from Healthy individuals, and for discriminating patients with Mild Cognitive Impairment from Healthy individuals (excluding Dementia cases). The 2.5^th^ and 97.5^th^ percentiles are also reported.*

| Scores | (MCI or Dem.) vs. Healthy | MCI vs. Healthy |
| --- | --- | --- |
| Raw | 0.9432 [0.940,0.946] | 0.9204 [0.916,0.924] |
| Corrected | 0.9247 [0.920,0.929] | 0.8952 [0.889,0.901] |
| Difference | 0.0185 [0.016,0.021] | 0.0253 [0.022,0.029] |

*Table e8: Average Area Under the Curve (AUC) in the validation sample across 10,000 simulations from the sensitivity analysis using the model from Equation 1 without rounding nor clipping, with a wider distribution (SD =3.4). The AUC is presented for raw and for corrected scores, for discriminating patients with cognitive impairment (Mild Cognitive Impairment or Dementia) from Healthy individuals, and for discriminating patients with Mild Cognitive Impairment from Healthy individuals (excluding Dementia cases). The 2.5^th^ and 97.5^th^ percentiles are also reported.*

| Scores | (MCI or Dem.) vs. Healthy | MCI vs. Healthy |
| --- | --- | --- |
| Raw | 0.9286 [0.925,0.932] | 0.9012 [0.897,0.906] |
| Corrected | 0.8942 [0.888,0.900] | 0.8555 [0.848,0.863] |
| Difference | 0.0344 [0.030,0.039] | 0.0457 [0.040,0.052] |

*Table e9: Average sensitivity and specificity in the validation sample across 10,000 simulations from the sensitivity analysis, using the model from Equation 1, with rounding and clipping, for three different marginal cutoffs. Values are presented for raw and for corrected scores for discriminating MCI patients from healthy individuals. The 2.5th and 97.5th percentiles are also reported.*

|  |  | Raw | | Corrected | |  |
| --- | --- | --- | --- | --- | --- | --- |
| Age | Edu. | Sensitivity [%] | Specificity [%] | Sensitivity [%] | Specificity [%] | Cutoff |
|  |  | 59.4 [48.9,61.9] | 96.4 [96.0,98.0] | 40.9 [37.3,44.4] | 97.7 [97.2,98.1] | Specificity = 97.7% |
|  |  | 86.5 [85.5,87.5] | 82.5 [82.1,82.9] | 77.9 [75.6,79.8] | 84.1 [82.9,85.2] | Specificity = 84.1% |
|  |  | 86.7 [85.4,91.7] | 82.1 [74.4,82.9] | 84.0 [80.4,87.4] | 77.7 [73.2,82.0] | Sensitivity = 84.1% |
| (54,59] | 17 | 6.7 [0.0,23.1] | 99.9 [99.8,100.0] | 37.3 [11.1,66.7] | 98.0 [96.2,99.1] | Specificity = 97.7% |
| (84,89] | 5 | 87.3 [78.8,90.6] | 72.8 [68.7,83.1] | 42.2 [38.2,46.1] | 97.5 [96.3,98.4] |  |
| (54,59] | 17 | 33.1 [10.0,58.3] | 98.6 [97.9,99.2] | 72.9 [47.8,94.1] | 87.3 [81.1,89.5] | Specificity = 84.1% |
| (84,89] | 5 | 98.7 [97.8,99.5] | 32.3 [29.2,35.4] | 79.9 [76.4,83.0] | 82.2 [79.5,84.9] |  |
| (54,59] | 17 | 33.7 [10.0,60.0] | 98.5 [96.7,99.2] | 82.7 [60.0,100.0] | 79.2 [73.3,81.8] | Sensitivity = 84.1% |
| (84,89] | 5 | 98.7 [97.7,99.7] | 31.8 [20.8,35.6] | 84.7 [78.0,90.1] | 76.2 [69.3,83.8] |  |

Edu = education

*Table e10: Average sensitivity and specificity in the validation sample across 10,000 simulations from the sensitivity analysis, using the model from Equation 2, with rounding and clipping, for three different marginal cutoffs. Values are presented for raw and corrected scores for discriminating MCI patients from healthy individuals. The 2.5^th^ and 97.5^th^ percentiles are also reported.*

|  |  | Raw | | Corrected | |  |
| --- | --- | --- | --- | --- | --- | --- |
| Age | Edu. | Sensitivity [%] | Specificity [%] | Sensitivity [%] | Specificity [%] | Cutoff |
|  |  | 56.7 [47.8,60.2] | 96.5 [96.0,98.0] | 40.6 [36.8,45.1] | 97.7 [97.2,98.1] | Specificity = 97.7% |
|  |  | 85.8 [82.8,89.9] | 81.3 [76.6,84.3] | 77.5 [75.4,79.8] | 84.1 [82.9,85.2] | Specificity = 84.1% |
|  |  | 86.7 [82.9,90.0] | 80.1 [76.6,84.3] | 84.0 [80.4,87.3] | 78.1 [72.9,82.0] | Sensitivity = 84.1% |
| (54,59] | 17 | 4.2 [0.0,18.2] | 100.0 [99.8,100.0] | 38.3 [13.3,64.3] | 98.0 [97.2,98.7] | Specificity = 97.7% |
| (84,89] | 5 | 89.1 [81.6,92.7] | 69.5 [64.1,80.1] | 43.9 [32.5,50.9] | 97.1 [95.5,98.9] |  |
| (54,59] | 17 | 30.7 [6.7,58.8] | 98.7 [97.4,99.6] | 76.9 [53.3,100.0] | 84.7 [82.7,86.6] | Specificity = 84.1% |
| (84,89] | 5 | 99.3 [98.4,100.0] | 23.8 [15.5,30.3] | 79.0 [71.3,85.5] | 82.4 [76.5,88.5] |  |
| (54,59] | 17 | 32.8 [7.7,61.5] | 98.5 [97.3,99.5] | 82.8 [60.0,100.0] | 78.4 [73.1,85.9] | Sensitivity = 84.1% |
| (84,89] | 5 | 99.4 [98.4,100.0] | 22.1 [15.3,30.1] | 84.3 [80.7,90.7] | 77.1 [67.1,81.1] |  |

Edu = education

*Table e11: Average sensitivity and specificity in the validation sample across 10,000 simulations from the sensitivity analysis, using the model from Equation 3, with rounding and clipping, for three different marginal cutoffs. Values are presented for raw and corrected scores, for discriminating MCI patients from healthy individuals. The 2.5^th^ and 97.5^th^ percentiles are also reported.*

|  |  | Raw | | Corrected | |  |
| --- | --- | --- | --- | --- | --- | --- |
| Age | Edu. | Sensitivity [%] | Specificity [%] | Sensitivity [%] | Specificity [%] | Cutoff |
|  |  | 57.9 [56.5,59.3] | 96.7 [96.6,96.9] | 40.4 [36.1,43.9] | 97.7 [97.2,98.1] | Specificity = 97.7% |
|  |  | 88.0 [84.2,91.1] | 80.1 [76.4,84.5] | 77.4 [74.8,79.6] | 84.1 [82.9,85.2] | Specificity = 84.1% |
|  |  | 86.4 [84.0,91.0] | 82.3 [76.5,84.5] | 84.0 [80.4,87.3] | 77.4 [73.1,81.4] | Sensitivity = 84.1% |
| (54,59] | 17 | 3.0 [0.0,15.0] | 100.0 [99.9,100.0] | 43.0 [16.7,70.0] | 97.3 [96.4,98.7] | Specificity = 97.7% |
| (84,89] | 5 | 81.8 [78.8,84.8] | 80.2 [77.5,82.9] | 42.6 [30.8,48.5] | 97.3 [95.9,99.0] |  |
| (54,59] | 17 | 25.9 [0.0,53.3] | 99.1 [98.3,99.8] | 80.0 [56.2,100.0] | 81.8 [79.2,89.0] | Specificity = 84.1% |
| (84,89] | 5 | 98.2 [96.4,99.5] | 35.5 [27.2,45.3] | 78.6 [69.5,84.3] | 82.8 [78.0,89.5] |  |
| (54,59] | 17 | 22.7 [0.0,50.0] | 99.4 [98.4,99.8] | 83.2 [60.0,100.0] | 77.9 [68.8,83.1] | Sensitivity = 84.1% |
| (84,89] | 5 | 97.8 [96.2,99.4] | 39.3 [27.8,45.7] | 83.5 [79.2,90.4] | 77.9 [68.2,82.6] |  |

Edu = education

*Table e12: Average sensitivity and specificity in the validation sample across 10,000 simulations from the sensitivity analysis, using the model from Equation 4, with rounding and clipping, for three different marginal cutoffs. Values are presented for raw and corrected scores, for discriminating MCI patients from healthy individuals. The 2.5^th^ and 97.5^th^ percentiles are also reported.*

|  |  | Raw | | Corrected | |  |
| --- | --- | --- | --- | --- | --- | --- |
| Age | Edu. | Sensitivity [%] | Specificity [%] | Sensitivity [%] | Specificity [%] | Cutoff |
|  |  | 56.3 [54.9,57.7] | 96.8 [96.6,96.9] | 41.2 [37.8,45.2] | 97.7 [97.2,98.1] | Specificity = 97.7% |
|  |  | 86.7 [85.8,87.7] | 81.5 [81.1,81.9] | 78.1 [76.3,80.1] | 84.1 [82.9,85.2] | Specificity = 84.1% |
|  |  | 87.0 [85.7,92.3] | 81.1 [71.9,81.9] | 84.0 [80.4,87.3] | 78.0 [73.6,82.1] | Sensitivity = 84.1% |
| (54,59] | 17 | 15.3 [0.0,36.4] | 99.7 [99.4,100.0] | 36.9 [12.5,63.6] | 98.2 [97.4,98.9] | Specificity = 97.7% |
| (84,89] | 5 | 74.8 [71.5,78.1] | 86.2 [83.9,88.4] | 42.1 [32.8,52.1] | 97.3 [95.3,99.0] |  |
| (54,59] | 17 | 50.3 [25.0,75.0] | 96.0 [94.9,97.0] | 75.7 [50.0,100.0] | 85.6 [83.7,87.4] | Specificity = 84.1% |
| (84,89] | 5 | 95.6 [94.0,97.1] | 52.2 [49.0,55.5] | 77.0 [71.9,85.8] | 84.2 [75.9,88.2] |  |
| (54,59] | 17 | 50.9 [25.0,76.9] | 95.8 [92.1,97.0] | 82.9 [60.0,100.0] | 78.4 [74.2,86.5] | Sensitivity = 84.1% |
| (84,89] | 5 | 95.7 [94.0,97.9] | 51.7 [38.9,55.5] | 84.6 [81.5,88.1] | 76.9 [72.3,80.0] |  |

Edu = education

*Table e13: Average sensitivity and specificity in the validation sample across 10,000 simulations from the sensitivity analysis, using the model from Equation 1 without rounding nor clipping, with a wider distribution for the residuals (SD =3.4), for three different marginal cutoffs. Values are presented for raw and corrected scores, for discriminating MCI patients from healthy individuals. The 2.5^th^ and 97.5^th^ percentiles are also reported.*

|  |  | Raw | | Corrected | |  |
| --- | --- | --- | --- | --- | --- | --- |
| Age | Edu. | Sensitivity [%] | Specificity [%] | Sensitivity [%] | Specificity [%] | Cutoff |
|  |  | 42.9 [39.4,46.4] | 97.7 [97.2,98.1] | 31.0 [27.6,34.4] | 97.7 [97.2,98.1] | Specificity = 97.7% |
|  |  | 79.6 [77.8,81.2] | 84.1 [83.0,85.3] | 69.2 [66.8,71.4] | 84.1 [82.9,85.2] | Specificity = 84.1% |
|  |  | 84.0 [80.4,87.4] | 79.6 [75.4,83.3] | 84.0 [80.4,87.3] | 69.2 [64.1,74.0] | Sensitivity = 84.1% |
| (54,59] | 17 | 4.2 [0.0,17.6] | 99.9 [99.8,100.0] | 31.0 [8.3,57.1] | 97.7 [96.7,98.6] | Specificity = 97.7% |
| (84,89] | 5 | 70.2 [65.3,74.9] | 83.4 [79.9,86.7] | 31.0 [25.8,36.4] | 97.7 [96.4,98.8] |  |
| (54,59] | 17 | 28.7 [7.1,53.8] | 98.1 [97.3,98.8] | 69.1 [42.9,92.3] | 84.1 [81.5,86.6] | Specificity = 84.1% |
| (84,89] | 5 | 95.6 [93.9,97.1] | 42.1 [38.2,46.0] | 69.2 [64.4,73.8] | 84.1 [80.7,87.2] |  |
| (54,59] | 17 | 35.7 [11.1,62.5] | 96.9 [95.2,98.2] | 83.9 [62.5,100.0] | 69.2 [62.9,75.0] | Sensitivity = 84.1% |
| (84,89] | 5 | 97.1 [95.3,98.6] | 34.5 [28.0,41.2] | 84.0 [79.4,88.4] | 69.2 [63.0,74.9] |  |

Edu = education

**References**

1. Istituto Nazionale di Statistica (2023) Resident population on 1st January. http://dati.istat.it/Index.aspx. Accessed 26 Apr 2023

2. Istituto Nazionale di Statistica (2022) Annuario statistico italiano 2021. https://www.istat.it/it/archivio/264305. Accessed 29 Apr 2023

3. De Ronchi D, Berardi D, Menchetti M, et al (2005) Occurrence of cognitive impairment and dementia after the age of 60: a population-based study from Northern Italy. Dement Geriatr Cogn Disord 19:97–105

4. Petersen RC, Lopez O, Armstrong MJ, et al (2018) Practice guideline update summary: Mild cognitive impairment: Report of the Guideline Development, Dissemination, and Implementation Subcommittee of the American Academy of Neurology. Neurology 90:126–35

5. Alzheimer Europe (2019) Dementia in Europe Yearbook 2019. https://www.alzheimer-europe.org/sites/default/files/alzheimer_europe_dementia_in_europe_yearbook_2019.pdf
